# Supplementary material for: Enhancement of perpendicular magnetic anisotropy and its electric field-induced change through interface engineering in Cr/Fe/MgO
Source: Sci Rep. 2017 Jul 20;7:5993. doi: 10.1038/s41598-017-05994-7 (PMC5519679; doi:10.1038/s41598-017-05994-7)
Supplement: Supplementary file 1 — Supplementary information [file 41598_2017_5994_MOESM1_ESM.pdf]

Supplementary information

Enhancement of perpendicular magnetic anisotropy and its electric field-induced change through interface engineering in Cr/Fe/MgO

A. Kozioł-Rachwał*[1,2], T. Nozaki[1], K. Freindl[3], J. Korecki[2,3], S. Yuasa[1] and Y. Suzuki[1,4]

[1] National Institute of Advanced Industrial Science and Technology, Spintronics Research Center, Tsukuba, Ibaraki 305-8568, Japan

[2] Faculty of Physics and Applied Computer Science, AGH University of Science and Technology, al. Mickiewicza 30, 30-059 Kraków, Poland

[3] Jerzy Haber Institute of Catalysis and Surface Chemistry, Polish Academy of Sciences, ul. Niezapominajek 8, 30-239 Kraków, Poland

[4] Graduate School of Engineering Science, Osaka University, 1-3 Machikaneyama, Toyonaka, Osaka 560-8531, Japan

Table 1 Hyperfine parameters derived from the numerical fits of the CEMS spectra for Cr/^{57}Fe/MgO before and after annealing. IS denotes the average value of the isomer shift with respect to α-Fe, B_{HF} is the average hyperfine magnetic field, ΔB_{HF} denotes the average Gaussian width of the B_{HF} distribution for a given site, and QS is the average quadrupole splitting.

**Component/ Subcomponent**	**IS[mm/s]**	**B_{hf}[T]/Δ B_{hf}[T]**	**ε or QS [mm/s]**	**RI [%]**
**before annealing**				
A/1	-0.21(3)	14.6(3)	-0.026(4)	4
A/2	-0.16(3)	18.2(2)	-0.026(4)	13
A/3	-0.12(3)	20.7(5)	-0.026(4)	8
A/4	-0.07(3)	23.4(7)	-0.026(4)	6
A/5	-0.03(3)	26.3(9)	-0.026(4)	5
A/6	0.00(4)	28(1)	-0.026(4)	9
A/7	0.04(4)	30(2)	-0.026(4)	20
A/8	0.07(4)	32.9(5)	-0.026(4)	12
A/9	0.11(4)	35.1(3)	-0.026(4)	8
B	0.61(4)	32.7(2)/4.2(3)	0.09(2)	12
P	-0.04(2)	4.4(1)	0	3
**after annealing**				
A/1	-0.08(4)	18.7(4)	-0.06(4)	9
A/2	-0.07(4)	20.7(6)	-0.05(4)	11
A/3	-0.05(5)	23.6(3)	-0.02(4)	13
A/4	-0.03(5)	26.4(6)	-0.01(4)	9
A/5	-0.01(5)	29.3(7)	0.01(5)	10
A/6	0.00(5)	31.5(8)	0.03(5)	12
A/7	0.02(5)	33.6(2)	0.05(5)	18
C	0.10(3)	26.0(2)/1.8(2)	0	17

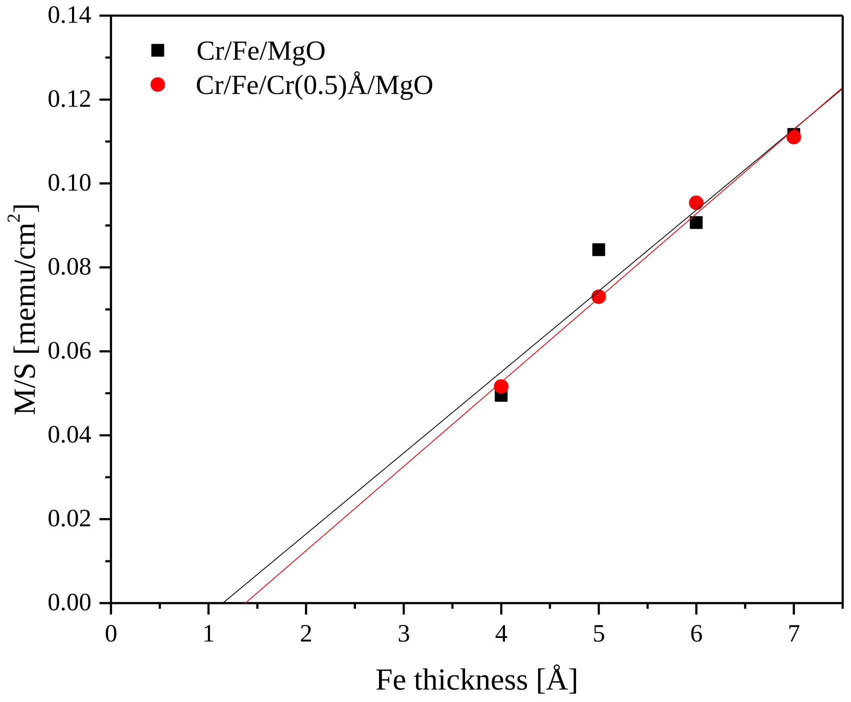

Figure 1a Areal magnetization dependence on Fe thickness obtained from SQUID measurements for Cr/Fe/MgO (black squares), and Cr/Fe/Cr/MgO with a Cr thickness of 0.5Å (red squares).

Table 2 Parameters derived from the fits of $K_{eff}(d_{Cr})$ dependence obtained for different Fe thicknesses.

t_{Fe} [Å]	curve used in fit	A[MJ/m^3]	B[MJ/(m^{3}Å)]	C[MJ/(m^{3}Å^2)]	d_{Cr}[Å]
4.9	A+B*d_{Cr}	0.75(8)	-0.3(2)	-	-
5.1	A+B*d_{Cr}	0.89(2)	-0.47(4)	-	-
5.3	A+B*d_{Cr}	0.91(2)	-0.54(4)	-	-
5.4	A+B*d_{Cr}+C*d_{Cr}^2	0.87(1)	0.0(5)	-0.56(6)	0.00(5)
5.6	A+B*d_{Cr}+C*d_{Cr}^2	0.92(3)	0.0(1)	-0.5(1)	0.00(1)
5.9	A+B*d_{Cr}+C*d_{Cr}^2	0.89(3)	0.8(3)	-1.5(4)	0.3(1)
6.1	A+B*d_{Cr}+C*d_{Cr}^2	0.89(3)	0.7(1)	-1.2(1)	0.3(1)
6.6	A+B*d_{Cr}+C*d_{Cr}^2	0.66(4)	1.5(3)	-1.5(4)	0.5(2)
